# The Impacts of COVID-19 Shock on Intergenerational Income Mobility: Evidence from China

**DOI:** 10.3390/ijerph191811546

**Published:** 2022-09-14

**Authors:** Shiqi Jiang, Lingli Qi, Xinyue Lin

**Affiliations:** 1 School of Economics and Resource Management, Beijing Normal University, Beijing 100875, China; 2 Energy Center, University of Auckland, Auckland 1010, New Zealand

**Keywords:** COVID-19, intergenerational income mobility, income inequality, sustainable society, economic shock, pandemic shock

## Abstract

The COVID-19 crisis has caused a huge negative shock to economic activities worldwide, leading to a reduction in income and changes in income distribution. Intergenerational mobility is an important indicator of sustainable social development. This paper explores the short-term impacts of the sudden COVID-19 pandemic on intergenerational income mobility and personal income in China. Using the variation in the number of confirmed cases across provinces, we construct a province-level pandemic intensity index and combine it with individual data from the China Family Panel Studies (CFPS). We apply a general difference-in-difference strategy to identify the causal effect of the pandemic on intergenerational income mobility. We find that personal income is positively related to parental income, and that the COVID-19 crisis has caused a decline in individual income and exacerbated intergenerational income persistence. A more intense COVID-19 pandemic shock is associated with a larger increase in intergenerational income elasticity and intergenerational income rank–rank slope. We found that with one standard deviation increase in local pandemic intensity, the intergenerational income elasticity increases by 0.315 and the intergenerational income rank–rank slope increases by 0.198 on average. The mechanism testing suggests that heterogeneous effects among different groups are the force underlying the results. Low-income, low-skilled, and low-parental-income individuals have suffered a more severe impact from the pandemic shock.

## 1. Introduction

The outbreak of COVID-19 has had a far-reaching detrimental influence on economies around the world, as seen by China’s GDP growth rate of 2.3% in 2020, which is far lower than the 6% growth rate that the IMF predicted in the absence of the pandemic [1]. In order to control the spread of the pandemic, many countries have adopted strict mandatory restrictions, such as social distancing, total lockdown and quarantine, as well as the control of production activities. These measures have further reduced domestic and international supply and demand, which in turn has severely damaged employment and personal income and worsened individual living standards [2,3,4]. According to China’s National Development and Reform Commission, in the first half of 2020, the per capita disposable income of Chinese residents fell by 1.3%. Among them, the income of urban residents fell by 2.0% and that of rural residents by 1.0%; in June 2020, the unemployment rate of China’s national urban survey was 5.7%, exceeding the same period in 2019 by 0.6% [5]. The structural changes in the economy triggered by COVID-19 and the growth in demand for public resources have exacerbated pre-existing inequalities [6]. The unequal impact of the COVID-19 pandemic on the employment incomes of different economic groups in society could exacerbate the inequality of opportunity and undermine social mobility [7].

Intergenerational mobility is an essential indicator of “equality of opportunity” in the economy and society, belonging to the category of social mobility and reflecting the dynamics of economic status between generations of a family [8]. Greater income inequality is associated with lower intergenerational income mobility [9]. Higher intergenerational mobility reflects “equality of opportunity” and is more conducive to stronger market vitality in a country or region, contributing to inclusive growth in an economy. On the other hand, low intergenerational mobility means that individuals’ achievements in society are closely tied to parental and family backgrounds and that social hierarchies are heavily entrenched, often leaving economies and societies without the competitive driving force for sustainable development, to the detriment of long-term economic growth [10,11]. The problem of income distribution inequality in China is extremely prominent and concerned. Compared with developed countries, China’s intergenerational mobility is low and tends to decline, and there is a risk of the further solidification of social classes [12]. Once the pandemic exacerbates household income and opportunity inequality, this greatly increases the pressure on China to maintain social equity, so this is an issue worthy of attention. However, direct evidence to analyse the impact of the COVID-19 pandemic on intergenerational income inequality is currently lacking, particularly in China.

This paper aims to examine the impact of the COVID-19 pandemic, a substantial adverse economic and social shock, on intergenerational income mobility. To establish the relationship between pandemic shocks and intergenerational income mobility, we follow the empirical design approach of Lou and Li [13] and refine it to fit our research problem. First, we use six rounds of the CFPS adult and household database from 2010 to 2020 to match income, education, and other demographic information for two generations of fathers/mothers and sons in a household. Then, we construct provincial-level pandemic shock indices using the number of infections to measure the intensity of pandemic shocks in different regions and combine them with micro-data to obtain six years of panel data. Furthermore, we devise a general difference-in-difference specification, using panel data rather than cross-sectional data, to study structural changes in income distribution caused by the pandemic. Cross-provincial differences in pandemics allow us to exploit the spatiotemporal variation in the COVID-19 pandemic shock, a quasi-global experiment, to identify the causal effects of pandemics on intergenerational income mobility.

Our baseline results show that the short-term shocks of the COVID-19 pandemic harmed the economy, resulting in a direct fall in individual incomes. The pandemic shock has reduced intergenerational income mobility and exacerbated social inequality. Provinces exposed to a stronger pandemic shock are associated with greater intergenerational income elasticities. With one standard deviation increase in local pandemic intensity at the margin, the intergenerational income elasticity increases by 0.315, and the intergenerational income rank–rank slope increases by 0.198 on average.

In addition, we find that parental income and parental income class are significantly positively associated with individual income, reflecting the general pattern. To address the endogeneity concern, we conduct a variety of robustness tests. First, we employ two measures of intergenerational income mobility, the intergenerational income elasticity and the intergenerational income rank–rank slope, and conduct regression analyses with two pandemic indices, the local index and the total index. Second, we conduct a placebo test, setting the assumed time of the COVID-19 pandemic outbreak before 2018, and do not find any similar caused effect. Finally, we consider potential household and provincial confounders to avoid estimation bias. All validity tests prove the robustness of our empirical outcomes. The further exploration of the mechanism shows that the reason for the decline in intergenerational income mobility caused by the COVID-19 pandemic is that the pandemic sharpens income inequality across social classes. The pandemic shock exacerbates income deterioration for individuals with a low income and low skills, and those from households in the lower income class.

The rest of this paper is as follows: Section 2 is a literature review. Section 3 introduces the empirical strategy. Section 4 describes the data used in our analysis. The results of the baseline model and robustness checks are shown in Section 5. Section 6 discusses the underlying mechanisms. Section 7 is the conclusion.

## 2. Literature Review

The COVID-19 pandemic and lockdown restrictions have been shown to have an enormously negative impact on the domestic and international economy and labour market [14,15,16]. The contraction of economic activity in both the supply and demand sectors reduces labour demand and incomes. The blockade further restricts labour mobility, creating a mismatch between supply and demand in the job market [17,18]. In earlier times, the COVID-19 pandemic had been touted by some as the “great equalizer” because it was indiscriminately contagious and restricted economic activity for almost everyone, regardless of their social status. However, current reality and research evidence refute this view, suggesting that the COVID-19 pandemic put a proportion of the population at a higher economic and health risk [19,20].

A growing body of literature examines the unequal impact of the COVID-19 pandemic on the labour market. Li et al. [21] used real-time recorded data and found that the pandemic has aggravated income inequality in Australia, increasing the Gini point by between 0.016 and 0.13 in April–June 2020 compared to February without policy support and worsening the living standards of low-income groups. With additional wage subsidies and welfare support from the government, the income inequality effect of the epidemic is eliminated. Evidence from Adams-Prassl et al. [15] using survey data from the UK, US, and Germany shows that COVID-19 shocks exacerbated income inequality within countries, and low-educated workers and women, who are more prone to being displaced, are most affected. Based on the analysis of data from a telephone interview survey of 31 developing countries, Bundervoet et al. [7] revealed that pandemic and lockdown policies have led to severe labour market contraction and reduced mobility in developing countries, resulting in approximately 36% unemployment and a 65% income decline, with a much more remarkable negative impact for urban and vulnerable groups, such as women, low-skilled non-farm wage workers, the self-employed, and the poor. While they suggest that intergenerational mobility may undergo a further decline since children from low-income families and areas suffer more academic losses and a higher rate of school drop-out due to the pandemic, they did not examine it directly. Additionally, COVID-19 exacerbates gender inequality in the labour market and income [22,23]. Not quite consistent with above conclusions that the largest declines appear in low-end workers, Campello et al. [24] find the largest declines in small companies and high-skilled jobs in the United States.

In the case of China, different occupational types of workers are also at unequal risk, and private, micro- and small enterprises, informal workers, and women are hit harder. The state-owned sector, large enterprises and formal workers, are retained, and small businesses are more likely to be pushed to the brink of collapse [25]. An analysis by Che et al. [17] suggests that the COVID-19 crisis has restricted population mobility, making it more difficult for migrant workers to obtain jobs than urban resident workers and exacerbating poverty among low-income people. Zhang et al. [26] used a computable general equilibrium (CGE) model to make a comprehensive assessment of the short-term impact of COVID-19 on the employment and income of different groups in China, finding that the pandemic lowered wages and exacerbated unemployment and poverty, as well as that female, low-skilled, and low-income groups were more vulnerable to pandemic shocks. However, the CGE model’s conclusions are from simulations rather than real changes in wages reported by individuals, and the macro-model is not applicable to analyse intergenerational problems.

The existing literature deduces that the pandemic has exacerbated domestic income inequality, both in developed and developing countries, by affecting the income and thus the well-being of different households and individuals [27,28,29]. Crossley et al. [30] found that COVID-19 has reduced the incomes of low-income households even further, with UK data indicating that nearly half of individuals have experienced a 10% drop in household income. Among those, the lowest 20% of the income distribution experience the largest decline. Using panel data from three sizeable representative population surveys in Germany between June and November 2020, Immel et al. [31] found that the COVID-19 pandemic has heightened concerns about health and unemployment among German residents: as of April, 5% of the respondents reported unemployment due to the pandemic. Self-employed persons, marginally employed workers, and low-income households experience a heavier burden. Qian and Fan [32] suggest that in China, where economic and social status favours individual resilience to the effects of COVID-19, the crisis has created new inequalities that urgently support policies in favour of disadvantaged groups. Luo et al. [33] use survey data of post-pandemic Chinese households to find that household income declines are more severe for poor families than for wealthy families, with self-reported results indicating that 23% of households lifted out of poverty are likely to fall back into it. While these studies are mainly based on post-pandemic survey data, we use multi-year data before and after COVID-19 to capture more information. Moreover, we are the first to directly measure its impact on household and intergenerational income inequality by matching children and parents. In addition, some countries are aware of the threat of the pandemic to increased poverty and have taken a variety of compensatory measures [21,34]. However, a report from UNDP [35] implies that social assistance policies are more effective in reducing poverty in higher-income countries, but not in low-income countries, given the insufficient amount of assistance.

Due to the significance of intergenerational mobility to the social economy, much research has been conducted on its determining factors; internal intergenerational income micro-transmission pathways are divided into “genetic” and “environmental”. The former refers to the innate talent that children obtain from their parents through inheritance, while the latter relates to parents’ investment in and cultivation of their children’s acquired growth environment [36]. The disparity in adult income stems from the fact that they are from families with different income levels and receive unequal investments in human capital as they grow up, and these differences in upbringing have a long-term, significant impact on the formation of people’s capabilities [37,38]. Due to different budget constraints, children from wealthy families receive a higher investment in human capital, are more likely to receive higher levels of education, and are more likely to find occupations with a higher economic and social status and earn higher incomes as they grow up [39,40,41]. In addition, children with higher family endowments also gain advantages from social capital, such as family social ties, making the children of relatively wealthier families more competitive in the labour market and enhancing the flow of resources to the elites [42,43,44].

The macro-economic and social environment and institutions play a defining role in intergenerational mobility. For example, labour market fragmentation and gaps in returns to human capital can increase income inequality and weaken intergenerational equity [45]. A study by Aiyar and Ebeke [11] using an internationally comparable data set demonstrates that unequal educational opportunities exacerbate income inequality and hinder intergenerational mobility, thereby widening the gap between the rich and the poor. It also shows that in societies where the negative effect of income inequality on economic growth is greater, intergenerational mobility is lower, while in regions with more remarkable economic and social growth, more equitable wealth distribution, outstanding social capital, educational opportunities, and stable family structures, intergenerational mobility tends to be higher [46,47,48]. Vijverberg [49] argues that a favourable macro-economic environment could promote social mobility. In 2018, the World Bank’s report, *Fair Progress? Economic Mobility across Generations around the World*, pointed out that intergenerational mobility is positively correlated with the level of economic development. However, the report indicates that intergenerational mobility in China declines as GDP per capita rises.

Based on the channels of family resource transmission and social instruction, we infer that the pandemic’s impact on intergenerational income mobility is related to the gap in the ability of families in different economic classes to withstand economic risks. Economically vulnerable low-income households struggled harder during the crisis than their more advantaged peers [32]. High-income households may be at lower risk of loss of income, as their members generally tend to have higher human capital and occupational status, allowing the use of broader social capital and various other means against crisis shocks [50].

A growing body of literature has studied the impact of exogenous shocks and policies on intergenerational mobility, but there is currently a lack of direct evaluation of the impact of COVID-19, and this study bridges this gap. Lou and Li [13] used micro-data to verify the effect of export shocks on intergenerational education persistence and mobility. They discovered that export expansion increases the earnings of low-skilled workers and improves the educational attainment of children from low-education households, leading to intergenerational educational mobility in China. Ahsan and Chatterjee [51] provide evidence on the impact of trade liberalization on occupational mobility in India, finding that high-skilled export shocks generate increasing demand for high-skilled labour and promote intergenerational occupational mobility. In addition, many papers have assessed the effects of public policies on promoting equal opportunities. Increasing government spending, especially on public services, and expanding the education supply can help narrow the gap between the rich and the poor and raise the likelihood of upward mobility for children from low- and middle-income families. This evidence is derived from China, the United States, Norway, and Denmark [37,52,53,54,55]. Other studies have explored the dynamics of social equity and intergenerational mobility under different economic and social systems, market-based transitions, and technological advances [12,56,57].

In general, this study contributes to the literature in at least three ways. First, our paper examines, for the first time, the effect of COVID-19 on intergenerational income mobility in China. Previous studies have examined the micro- and macro-level factors influencing intergenerational income mobility, and a growing body of literature focuses on the unequal consequences of COVID-19. However, since the pandemic hit society, little has been discovered regarding the impact of the pandemic, a negative economic shock, on intergenerational mobility in China, particularly in terms of empirical evidence. We innovatively explore the impact of the crisis on household welfare and social equity measured by income distribution across generations to enrich the evidence on the COVID-19 pandemic’s socioeconomic consequences. Second, this paper innovatively uses years of micro-survey data and the spatiotemporal differences of the pandemic to identify the direct effects of the pandemic on income distribution and intergenerational mobility, in contrast to the existing literature, which primarily uses numerical simulation methods and personal survey data after the pandemic. Finally, this research contributes to the micro-econometric literature on the effects of exogenous economic shocks on intergenerational mobility. Our analysis examines how the pandemic has affected intergenerational income mobility in China through an inequal shock on income.

## 3. Empirical Strategy

### 3.1. Intergenerational Income Mobility

The baseline empirical model of intergenerational income mobility, as shown in Equation (1), is based on the theoretical framework proposed by Becker and Tomes [42].

(1) $$Y_{i}^{c}=\beta_{0}+\beta_{1}Y_{i}^{f}+\gamma u_{i}^{c}$$

Suppose that family *i* consists of a parent *f* and a child *c*, and the total household income is the parent’s income $Y_{i}^{f}$. The household income $Y_{i}^{f}$ needs to be allocated between the parent′s consumption $C_{i}^{f}$ and the investment in the child’s human capital *I_i_* to maximize household utility (assuming a Cobb–Douglas form). The child’s income $Y_{i}^{c}$ depends on their human capital investment *I_i_* and investment rate of return *r*, as well as a generalized endowment $u_{i}^{c}$, including luck, which is generally considered to be randomly distributed and can also be regarded as a random disturbance term. The economic model of this intergenerational income transmission mechanism is expressed as follows:

(2) $$Max\;U_{i}\left(C^{f},Y^{c}\right)=\left(1-\lambda \right)logC_{i}^{f}+\lambda logY_{i}^{c}$$

(3) $$s.t.\;Y_{i}^{f}=C_{i}^{f}+I_{i}$$

(4) $$Y_{i}^{c}=\left(1+r\right)I_{i}+u_{i}^{c}$$

The equilibrium condition for maximizing utility is:

(5) $$\left(\partial U/\partial C^{f}\right)/\left(\partial U/\partial Y^{c}\right)=1+r$$

The optimal solution is:

(6) $$Y_{i}^{c}=\beta Y_{i}^{f}+\gamma u_{i}^{c}$$

where the coefficient $\beta =\lambda \left(1+r\right)$ indicates the magnitude of the intergenerational income correlation; the larger the β, the lower the intergenerational income mobility. Regressions are often carried out using the logarithmic form of income rather than the horizontal value [58]. Therefore, Equation (1) is rewritten as follows:

(7) $$lnY_{i}^{c}=\beta_{0}+\beta_{1}lnY_{i}^{f}+\gamma u_{i}^{c}$$

We can see that the influence degree of the parent’s income on the child’s income is $\partial Y_{i}^{c}/\partial Y_{i}^{f}=\beta$. Therefore, *β*, also known as intergenerational income elasticity (IGE), is a commonly used indicator in economics to express the magnitude of intergenerational income mobility [59]. The IGE measures the relative change in the income of two generations, and its value represents the percentage change in the income of the child caused by each 1% change in the parent’s income. The coefficient is generally positive because the child’s income tends to be positively correlated with their parent’s income. Our baseline empirical specification is a variant extension of Equation (7), which is detailed in the following section.

Another measure of the correlation between child and parental income is the intergeneration income rank–rank slope, which indicates the correlation between a child’s income at the cohort-rank percentile and parental income at the cohort-rank percentile, also known as income class mobility [48]. The equation relating the income classes of the child and the parent is deformed based on Equation (1) as follows:

(8) $$Rank_{i}^{c}=\alpha_{0}+\alpha_{1}Rank_{i}^{f}+\varphi v_{i}$$

where $\alpha_{1}$ is the estimated income rank–rank slope, the magnitude of which indicates the rank of the children’s income caused by a one-rank increase in the parent’s income. The larger the value, the greater the intergenerational income rank correlation and the lower the intergenerational income mobility. The intergenerational income mobility measured by the income rank–rank slope in this study can be used as a robustness test for the IGE. The income rank–rank slope only uses information on the ranking of parental and child permanent income for intergenerational mobility estimation, which improves the lifetime error measure on intergenerational income elasticity estimates to some extent. However, the income rank–rank slope is not as intuitive as IGE.

### 3.2. Regression Specification

This section details our identification strategy linking the COVID-19 pandemic to intergenerational income mobility. As a natural experiment, COVID-19 was an unpredictable event, so we constructed a general difference-in-difference setting. If we obtained information on the income of individuals and their parents before (pre-experiment) and after (post-experiment) the pandemic, we could obtain changes in intergenerational income mobility across time. However, for individuals exposed to the pandemic simultaneously in a country, we identified spatial variation across provinces by constructing an indicator of the pandemic shock intensity at the provincial level to determine inter-group differences.

We extended and refined Equation (7) by including pandemic shock variables to identify the impact of the pandemic on intergenerational income mobility. Thus, the baseline model for this study is shown in Equation (9):

(9) $$lnY_{ipt}^{c}=\beta_{1}lnY_{ipt}^{f}+\beta_{2}COVID_{p}\times T+\beta_{3}(COVID_{p}\times T)\times lnY_{ipt}^{f}+\gamma_{1}X_{i}+FE_{t}+FE_{p}+\varepsilon_{ipt}$$

where $lnY_{ipt}^{c}$ and $lnY_{ipt}^{c}$ denote the income of an individual *i* from a given household and their parents’ generation, respectively, and $\beta_{1}$ is the intergenerational income elasticity in the absence of COVID-19 shock, and in general, $\beta_{1}$ is expected to be positive; *T* is used to identify the treatment period, and only 2020 represents post-treatment, so *T* = 1 if survey year = 2020, and *T* = 0 if survey year < 2020.

$COVID_{p}$ is and indicates the intensity of the pandemic shock to the province *p*. $COVID_{p}\times T$ is the interaction term between the pandemic intensity index and the treatment period dummy variable, identifying the pandemic shock treatment to which individuals are subjected. The coefficient of the interaction term between the treatment variable and the log of parental income, $\beta_{3},$ is the most interesting parameter, measuring the average treatment effect of the pandemic shock on intergenerational income elasticities. A positive $\beta_{3}$ indicates that the pandemic shock reduces intergenerational income mobility and strengthens the link between a child’s income and parental income. The model was also set up to capture the effect of the pandemic shock on the income of children, as measured by $\beta_{2}$, and we expected the pandemic to have had a negative impact on the income of individuals, as the pandemic brought down the overall economy. In addition, we included two-way fixed effects in the model; *FE_t_* is a time fixed effect that controls for the time trend inherent around the treatment period, and *FE_p_* is a province fixed effect that includes province characteristics that do not change over time. Two-way fixed effects eliminate systematic heterogeneity between years and provinces, and we also used clustering robust standard deviations at the provincial level to correct for intra-provincial autocorrelation.

## 4. Data

### 4.1. Sample Construction

The micro-individual data in this paper are based on the China Family Panel Studies (CFPS) conducted by the Institute of Social Science Survey (ISSS) of Peking University. Through panel surveys of nationally representative sample villages, families, and individuals, CFPS can reflect the basic patterns of China’s economic development and social changes and provide micro-data statistics for academic research and policy analysis. The CFPS data cover nearly all aspects of China’s economic, social, demographic, and educational changes and include statistical data on income and intergenerational relations for this study. In particular, the CFPS 2010 baseline survey was conducted on a sample of household members from 25 provinces in China, covering approximately 95% of the country’s population. To track relocated sample households, the follow-up surveys in 2012, 2014, 2016, 2018, and 2020 were extended to 31 provinces. We used the information on family relationships, adult income, and other demographic characteristics in CFPS to analyse intergenerational income mobility changes through matching parents and their children.

CFPS2020 is the most recent nationally representative publicly available household and individual survey database in China, and covers the year affected by COVID-19. In addition, CFPS2010–2020 data have the advantage of being multi-year and containing information on two generations in a family, allowing us to capture the short-term impact of the pandemic shocks on income distribution patterns by identifying the pre- and post-pandemic differences, which is one of the bases of our empirical strategy.

Based on the purpose of this paper, the CFPS data were further processed; using individual codes, family codes, and family relationship codes, fathers and mothers in the same household were matched with sons or daughters. The samples only contained children that could match their parents’ generation. Children that were younger than 20 years old or older than 50 years old were excluded. Parents younger than 35 years old or older than 65 years old were excluded. We also excluded the observations whose age difference between parents and children were less than 15 years and those in school at the time of the survey. The potential life-cycle bias was reduced by restricting children’s and parents’ ages as close to lifetime earnings as possible. In this paper, six rounds of the CFPS adult database for 2010, 2012, 2014, 2016, 2018, and 2020 were matched for parent and child generations and later appended into panel data. Moreover, in the empirical regressions, we excluded individuals that missed key information, such as income, age, and education level, and finally obtained a total of 6053 matched parent–child pairs. Of these, 1482, 2194, 1535, 96, 418, and 328 parent–child pairs were matched in 2010, 2012, 2014, 2016, 2018, and 2020, respectively.

### 4.2. Main Variables

The following variables were selected for this paper to analyse the impact of the COVID-19 shock on intergenerational income flows:

*Income*: The total personal annual income recorded in the CFPS survey was taken as the income variable. To ensure comparability between different years, the individual income was deflated according to the CPI of the corresponding year and expressed as the price level in 2010. Moreover, the income data below 1% and above 99% were winsorized to ensure the robustness of the regression results. We used the highest income of the father or mother as the parental income. Both the children’s and parents’ income were divided into 20 equal points within the same generation as income rank variables, increasing from 1 to 20.

*Control variables*: In addition to the core income variables, relevant individual and household characteristics that affect individual income levels needed to be included in the model as control variables. According to the Mincer equation, personal income levels were mainly influenced by factors such as human capital and work experience [41]. Therefore, based on the existing research, we introduced individual age, age squared, gender, years of schooling, type of household registration (urban or rural), whether they work in non-farm jobs, and parents’ age as control variables in the model. The control variables for both individuals and households were derived from the CFPS.

*Pandemic intensity index*: The key treatment variable in this paper was the intensity of the pandemic shock to provinces, which was measured mainly by the number of confirmed cases in each province. However, factors such as the local population and the potential extent of infection vary across regions. Using only the number of confirmed cases to measure the severity of the pandemic can be somewhat biased. Therefore, it is necessary to convert the absolute indicator of the number of confirmed cases into a relative indicator. This paper used the location entropy method to construct the province-level relative pandemic intensity index. The calculation is shown in Equation (10):

(10) $$COVID_{p}=\frac{Covidcase_{p}/Population_{p}}{Covidcase_{N}/Population_{N}}$$

where $COVID_{p}$ refers to the pandemic intensity index of province *p*. $Covidcase_{p}$ indicates the cumulative number of confirmed cases in province *p* up to 31 May 2020. (CFPS conducted surveys from June to August in each survey year, and the latest wave was from June to August 2020, so the cumulative number of confirmed COVID-19 cases before 31 May 2020 was used to construct the pandemic intensity indicators.) $Population_{p}$ refers to number of resident populations in province *p* at the end of 2019. $Covidcase_{N}$ denotes the cumulative confirmed cases nationwide up to 31 May 2020. $Population_{N}$ represents the total population of China at the end of 2019. A higher value for this indicator indicates a higher relative number of infections in the region, reflecting the more severe impact of the pandemic in the area.

**Figure 1:**
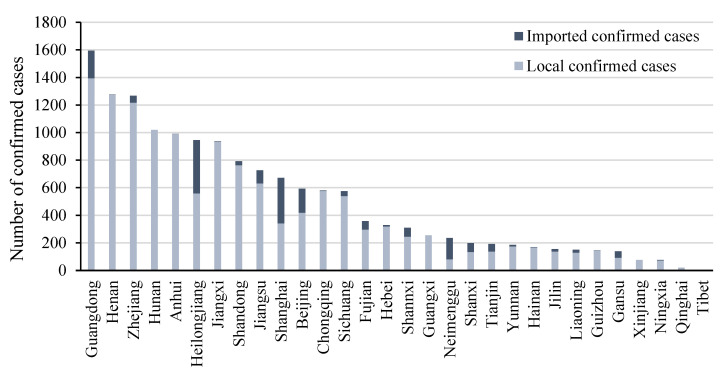
The cumulative numbers of COVID-19 confirmed cases in Chinese provinces as of May 2020.

**Figure 2:**
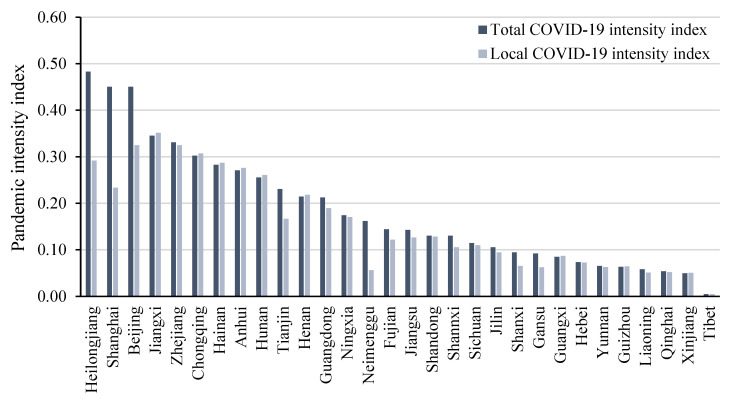
The pandemic intensity index of Chinese provinces.

The number of confirmed COVID-19 infections in each province was compiled from the official announcements of each province on the internet, as described in Figure 1. The year-end population data for each region were obtained from *the China Statistical Yearbook 2021*. This paper used COVID_L to represent the local pandemic index, which includes only the cumulative number of Chinese confirmed cases. COVID_T is the overall pandemic index, which consists of the cumulative number of Chinese confirmed cases in the local area and those imported from abroad. Figure 2 shows the pandemic intensity index of each province. Hubei province, not shown in Figure 1, was the worst-hit province of China in the early stage of the pandemic spread and had 68,135 confirmed cases as of May 2020. Hubei province is also excluded in Figure 2 and shows the most extensive pandemic intensity index of 19. In the short term, the COVID-19 shock measured by the number of confirmed cases was a near-exogenous shock, which is the basis for the validity of our estimates. We examined the correlation between the provincial pandemic index and the main socioeconomic variables. The results are reported in Table A1, and the coefficients of all economic variables are not significant. In the robustness test in Section 5.3, we further controlled for provincial characteristics to eliminate possible endogeneity.

The descriptive statistics of the main regression variables are shown in Table 1. There is a total of 6053 individual observations. Except for parental income, as well as the fathers’ and mothers’ ages, the rest are individual characteristic variables of children. The sample excludes Hainan province, so the pandemic intensity index in the province variables refers to the 30 provinces involved in China. In our analysis sample, the children’s ages ranged from 20 to 46 years old, with an average age of approximately 27; the fathers’ and mothers’ ages ranged from 36 to 65 years old, with an average age of 53 and 51, respectively. The age of parents in our sample was close to the stable income period of working life. The average income of the children’s generation of 20,203.15 was significantly higher than the average income of the fathers’ generation of 14,537.95, reflecting the absolute upward mobility of the income level of the children’s generation relative to the fathers’ generation over the period 2010–2020. The sample was dominated by male, rural, and non-farm worker groups.

## 5. Main Results

### 5.1. Baseline Results

The baseline empirical results reported in Table 2 provide direct evidence that the COVID-19 pandemic has hindered intergenerational income mobility and exacerbated social inequality. In columns (1) to (4) of Table 2, the estimated coefficients of the log of parental income and parental income rank, which represent intergenerational income elasticities and income rank–rank slope, both reveal statistically significant positive effects. This indicates that children’s income is positively related to parental income and that their income class within the same generation is closely associated with parental income class across parental generations. We are most concerned that the interaction term between the log of parental income and the pandemic shock treatment variable and the interaction term between parental income rank and the pandemic shock treatment variable are both negative and statistically significantly different from zero at the 1% confidence level, suggesting that exposure to a more severe pandemic shock increases the dependence of children on their father’s income in China.

We further explain the magnitude of the regression estimators. For example, in column (1) of Table 2, the size of the estimated coefficient for the interaction term between the log of parental income and the pandemic shock treatment variable is 0.089, with a robust standard deviation of 0.028; that is, one standard deviation increase in the local pandemic intensity index leads to an average increase of 0.315 in the intergenerational income elasticity compared to that without the pandemic, implying a decrease in intergenerational income mobility. In column (3), the estimated coefficient of the interaction term between the parental income rank and the pandemic shock treatment variable is 0.056, with a robust standard deviation of 0.009; that is, one standard deviation increase in the pandemic intensity index leads to an average increase of 0.198 in the intergenerational income rank–rank slope, which increases intergenerational income class persistence. In addition, the coefficient of the pandemic shock treatment variable is significantly negative in all four columns, suggesting that the pandemic has caused a direct decline in personal income and that the more severe the exposure to the pandemic, the greater the reduction in personal income.

In addition, we excluded individuals who were not working at the time and found that the regression results were similar to the baseline results, as shown in Table A2. We also conducted a robustness regression using the pandemic intensity index calculated by cumulative confirmed cased data as of 30 June and 31 July 2020. The results shown in Table A3 suggest the consistent conclusion that the pandemic shock has reduced intergenerational income mobility.

### 5.2. Robustness Checks: Placebo Tests

To test whether the impacts on intergenerational income elasticities are due to the COVID-19 pandemic shock, we conducted a placebo test to check the validity of our identification. This study’s placebo test is based on the assumed onset of COVID-19 pandemic shock. The counterfactual placebo test is an effective means of inferring causal effects. In fact, China was not affected by the pandemic until 2019, and based on this, we conducted a counterfactual analysis.

We removed the observations from CFPS2020 and retained only CFPS2010 to CFPS2018 to form our sample of the placebo test. We artificially assumed two scenarios: Scenario 1 (S1) assumes that the pandemic occurred in 2018, as shown in columns (1) and (2) of Table 3. Scenario 2 (S2) assumes that the pandemic occurred in 2016, at which point both 2016 and 2018 were assumed to be treated years, as shown in columns (3) and (4) of Table 3. Thus, we expected that the pandemic shock treatment variable, which reduced intergenerational income mobility shown in the baseline regression, would not display any effect on intergenerational income mobility for pre-2020 data in the placebo test. If our empirical setting was reasonable, the estimated parameters of the DID interaction terms were expected to be statistically insignificant or close to 0.

The placebo test results for Table 3 show that the interaction between the pandemic shock treatment variable and the log of parental income is statistically insignificant, and the interaction between the pandemic shock treatment variable and parental income rank is almost negligible. The results are consistent with our expectations, indicating the validity of our identification strategy. At the same time, the income of the younger generation is still significantly and positively correlated with the parental income, and the coefficient’s magnitude is similar to the baseline regression results.

In contrast, in columns (2) and (4) of Table 3, pandemic shock treatment variables are significantly larger than zero. This suggests that individual incomes increase over time in the absence of pandemic shocks and possibly because places that are more severely hit by epidemics tend to have higher levels of economic development. This is further supported by the fact that pandemic shocks in Table 2 directly lead to a decrease in personal income.

### 5.3. Robustness Checks: Family- and Province-Level Confounders

Some characteristics of individuals and households may affect intergenerational income elasticities, and if these effects were confounded in our identification, there is a risk that the estimates may be biased. First, because household resources are not equally distributed between sons and daughters, there is the potential for differences in income transmission. Therefore, in Table 4, we included an interaction term between the child’s gender and the log of parental income in column (1) as an additional control. Second, the pandemic shock may have also affected parental income, but their household and education levels were mainly unaffected. Thus, we added the interaction terms between the father’s household type (urban or rural), the father’s years of schooling, and parental logarithmic income in columns (2) and (3), respectively, to strip out the part of the intergenerational income elasticity that is affected by the household resident characteristics and parental education level channels. Third, there is a concern that region-specific characteristics, such as socioeconomic status, may impact intergenerational mobility and interfere with the estimation of crucial parameters, resulting in endogeneity. We included an interaction term between province-level GDP in 2019 and the log of parental income in column (4). In column (5), an interaction term between the proportion of the migrant population outside the province in 2020 and parental logarithmic income is presented. In column (6), we incorporated all the confounding factors into the regression.

From column (1) to column (6), we found that the regression results controlling for household and province confounders were consistent with the findings of the baseline regression results. The estimated coefficients of interaction terms between the log of parental income and pandemic shock treatment variables remain significantly positive with a similar size. Meanwhile, the coefficients of the pandemic shock treatment variables are similarly statistically significant and negative. This confirms that the estimates from our baseline regression are robust to the fact that pandemic shocks reduce intergenerational income mobility and increase income inequality in China.

## 6. Underlying Mechanism

The decline in intergenerational income mobility may result from a disproportionate decline in income among low-income groups due to the COVID-19 pandemic shock or an increase in income among high-income groups. Based on the available research and anecdotal evidence, we expect that the former is predominant and that the slowdown in intergenerational income mobility is attributable to the heterogeneity of income declines across higher- and lower-income groups caused by the pandemic shock. Income deterioration is more severe in the low-income group, which is more likely to come from low-income households. To test whether the pandemic worsened income distribution inequality, we first examined the impact of the COVID-19 pandemic shock on people’s incomes at different income levels. The results are shown in columns (1) and (2) of Table 5. There was individual income deterioration, and the interaction term between the children generation’s income rank and the pandemic shock variable was significantly positive, indicating that, as individual income classes rise, they can resist the fall in income caused by the pandemic shock.

Second, we examined the effect of the pandemic shock on the income of people in different parental income ladders, and the results are reported in columns (3) and (4) of Table 5. The interaction term between parental income rank and the pandemic shock variable remains significantly larger than zero. The negative effect of the pandemic shock on income is offset by this, implying that the income of individuals from low-income households is affected by the pandemic shock to a greater extent. Columns (5) and (6) report the association between the pandemic’s income shock and individuals’ educational level. The interaction term between years of education and the pandemic shock treatment variable is also significantly positive, with the magnitude of the reduction in individual income slowing down as the level of education increases.

The results shown in columns (1) to (6) of Table 5 suggest that pandemic shocks reduce intergenerational mobility due to widening income gaps. The negative effects of the pandemic shock are more pronounced for low-income and low-skilled groups, thus increasing intra- and intergenerational income inequality. The pandemic has caused an absolute economic downturn and changed the relative income distribution structure, damaging social stability and economic sustainability. Social equity must be considered in the process of economic recovery in the post-pandemic era.

## 7. Conclusions and Discussion

The sudden COVID-19 pandemic has posed unprecedented challenges for the economies of various countries, and China’s social economy has also been severely impacted. The health threat of the epidemic and the government’s unavoidable control policies have exacerbated supply contraction, reduced demand, a sluggish labour market, and a decline in personal income. However, there are differences in the impact of the pandemic on individuals from different social classes. This study combines several years of CFPS micro-individual data with provincial pandemic shock indices to provide direct empirical evidence of concerns about social inequality under the COVID-19 crisis.

We found that COVID-19 shocks have directly reduced individual income and exacerbated the dependence between individual and household income, reducing intergenerational income mobility. We also found that pandemic shocks primarily reduce intergenerational income mobility by reducing income among individuals with low income levels and low education levels, and form low-income households. In contrast, higher-income and educated individuals are more resilient to risk. Since the data used in this analysis are from May 2020, when the pandemic had only been around for approximately five months, this paper can only identify the impact of the pandemic shock in the short term and cannot capture its longer-term effects. Despite the data and scope restrictions on further research mentioned above, the findings of this study have important policy implications. The negative economic shock of the COVID-19 pandemic is structural and, in particular, has also exacerbated intra- and intergenerational inequalities in society. Policymakers must recognize the social inequalities caused by pandemic shocks and apply stimulus measures to boost the economy and compensate people for income losses.

Appropriate policy measures may be taken to mitigate the loss of income of the vulnerable population and the inequality caused by the pandemic. Especially for the poor and vulnerable, tilt policy can be used to promote inclusive recovery and their future risk resilience [60]. For example, first, the government should strengthen targeted assistance policies for low-income and vulnerable families severely affected by the pandemic. Additional income and welfare support can be used to counter the losses of low-income households, to guarantee their essential needs of living, food, housing, employment, health care, and education, so as to prevent further expansion of inequality. Public investment in specific health and education measures for vulnerable groups is necessary. The pandemic has had disproportionate impacts on access to education for poor children [7,18]. Before COVID-19, an individual’s family class, wealth, and educational availability, which determine their future career and income, are already dominant factors contributing to inequality of opportunity in developing countries [44]. This requires the provision of educational opportunities to children from disadvantaged families to reduce inequality of opportunity across income groups in the long term and prevent excessive intergenerational income persistence [7]. Second, the government and society should improve the unemployment assistance system and improve a fair employment environment. Livelihood assistance and unemployment benefits can be provided to low-income unemployed households. This also requires helping re-employ those who lost their jobs during the pandemic, with special support for vulnerable workers through appropriate active labour market policies. Third, policy preferences should be given to the sector directly affected by the pandemic and micro- and small enterprises (MSEs), reducing their financing costs and stimulating the market demand for labour. MSEs can take on a large amount of labour and absorb more low-income and low-skilled workers than large enterprises and state sectors. However, MSEs are less able to resist risks in crises [25]. Therefore, additional support can be given to prevent MSEs from unemployment and going bankrupt.

In conclusion, the evidence presented in this paper for China is also worthy of consideration by other countries. The increased social inequity caused by the pandemic shock, the consequent impact on sustainable economic and social development, and the long-term welfare of individuals are matters that require further attention from researchers and policymakers. There is room for further expansion under the research theme of this paper. One is that the long-term effects of the pandemic shock on education, employment, income inequality, and individual welfare can be examined after a long period of observation. Furthermore, the impact of China’s economic recovery policies and countermeasures on inequality could be further explored. These issues are important as China strives to achieve its goals of common prosperity.

## Figures and Tables

**Table 1:** Summary statistics of main variables.

	Mean	Std.	Min	Max
*Individual variables*				
Income	20,203.15	21,192.95	0	163,061.3
Income rank (IR)	12.082	6.837	1	20
Parental income	14,537.95	18,572.65	0	164,731.1
Parental income rank (IR)	8.719	6.809	1	20
Age	26.822	4.846	20	46
Male	0.702	0.457	0	1
Rural	0.738	0.440	0	1
Schooling year	10.472	4.084	0	22
Farm work	0.249	0.433	0	1
Father’s age	53.259	5.842	37	65
Mother’s age	51.615	5.730	36	65
*Province variables*				
COVID_L	0.799	3.538	0.005	19.526
COVID_T	0.814	3.460	0.005	19.122

**Table 2:** The effects of COVID-19 shock on intergeneration income mobility.

	ln (Child’s Income)		Income Rank	
	(1)	(2)	(3)	(4)
ln (parental income)	0.074 ***	0.074 ***		
	(0.016)	(0.016)		
COVID_L × T	−0.946 ***		−1.007 ***	
	(0.312)		(0.136)	
ln (parental income) × COVID_L × T	0.086 ***			
	(0.028)			
COVID_T × T		−1.007 ***		−1.040 ***
		(0.364)		(0.170)
ln (parental income) × COVID_T × T		0.092 ***		
		(0.033)		
Parental income rank			0.051 ***	0.051 ***
			(0.014)	(0.014)
Parental IR × COVID_L × T			0.056 ***	
			(0.009)	
Parental IR × COVID_T × T				0.059 ***
				(0.011)
Constant	6.422 ***	6.423 ***	2.500	2.506
	(0.807)	(0.807)	(1.747)	(1.747)
Individual controls	Yes	Yes	Yes	Yes
Province FE	Yes	Yes	Yes	Yes
Year FE	Yes	Yes	Yes	Yes
R-squared	0.557	0.557	0.508	0.508
Observations	6052	6052	6052	6052

Robust standard errors are shown in parentheses; ***, **, and * denote significance at the 1%, 5%, and 10% confidence levels respectively. In all regressions, robust standard errors are clustered at the province level. Individual controls, province fixed effect, and survey year fixed effect are included in all regressions. Estimated coefficients for individual control variables are consistent with empirical expectations and are, therefore, not reported in the results for the sake of brevity.

**Table 3:** Placebo tests.

	S1: Assumed COVID-19 Year 2018		S2: Assumed COVID-19 Year 2016–2018	
	ln (Income)	Income Rank	ln (Income)	Income Rank
	(1)	(2)	(3)	(4)
ln (parental income)	0.074 ***		0.074 ***	
	(0.016)		(0.016)	
COVID_L × T	0.018	0.188 **	1.843	0.215 **
	(0.240)	(0.074)	(1.644)	(0.095)
ln (parental income) × COVID_L × T	0.001		−0.171	
	(0.023)		(0.158)	
Parental income rank		0.049 ***		0.049 ***
		(0.014)		(0.014)
Parental IR × COVID_L × T		−0.010 *		−0.010
		(0.005)		(0.006)
Constant	6.705 ***	4.071 **	6.711 ***	4.077 **
	(0.848)	(1.714)	(0.847)	(1.714)
Individual controls	Yes	Yes	Yes	Yes
Province FE	Yes	Yes	Yes	Yes
Year FE	Yes	Yes	Yes	Yes
R-squared	0.554	0.526	0.554	0.526
Observations	5724	5724	5724	5724

Robust standard errors are shown in parentheses; ***, **, and * denote significance at the 1%, 5%, and 10% confidence levels respectively. In all regressions, robust standard errors are clustered at the province level. Individual controls, province fixed effect, and survey year fixed effect are included in all regressions. The table only reports the results of the local COVID-19 pandemic intensity index. The estimated results of the key interaction terms of the regression using the total COVID-19 pandemic intensity index are similar, as shown in Table A4.

**Table 4:** Robustness checks include family- and province-level confounders.

	Univariate Confounder Regression					Multivariate
	(1)	(2)	(3)	(4)	(5)	(6)
ln (parental income)	0.066 ***	0.052 ***	0.095 ***	0.104 ***	0.096 ***	0.100 ***
	(0.020)	(0.017)	(0.022)	(0.019)	(0.016)	(0.026)
COVID_L × T	−0.949 ***	−0.860 **	−1.037 ***	−0.919 ***	−0.917 ***	−0.932 ***
	(0.315)	(0.327)	(0.311)	(0.307)	(0.315)	(0.316)
ln (parental income) × COVID_L × T	0.086 ***	0.078 **	0.094 ***	0.084 ***	0.083 ***	0.085 ***
	(0.028)	(0.030)	(0.028)	(0.027)	(0.028)	(0.029)
ln (parental income) × male	0.011					0.013
	(0.016)					(0.017)
ln (parental income) × father’s hukou		0.029 **				0.016
		(0.012)				(0.011)
ln (parental income) × father’s edu year			−0.003 **			−0.003 *
			(0.002)			(0.001)
ln (parental income) × GDP_2019				−0.000 *		−0.000
				(0.000)		(0.000)
ln (parental income) × migration rate					−0.003 **	−0.002 *
					(0.001)	(0.001)
Constant	6.471 ***	6.520 ***	6.501 ***	6.415 ***	6.454 ***	6.640 ***
	(0.813)	(0.821)	(0.758)	(0.817)	(0.831)	(0.814)
Individual controls	Yes	Yes	Yes	Yes	Yes	Yes
Province FE	Yes	Yes	Yes	Yes	Yes	Yes
Year FE	Yes	Yes	Yes	Yes	Yes	Yes
R-squared	0.557	0.557	0.566	0.558	0.558	0.566
Observations	6052	6044	5871	6052	6052	5863

Robust standard errors are shown in parentheses; ***, **, and * denote significance at the 1%, 5%, and 10% confidence levels respectively. In all regressions, robust standard errors are clustered at the province level. Individual controls, province fixed effect, and survey year fixed effect are included in all regressions. The GDP of each province in 2019 is from *The China Statistical Yearbook 2020*. The table only reports the regression results of the epidemic index constructed by local confirmed cases. The table only reports the results of the local COVID-19 pandemic intensity index. The estimated results of the key interaction terms of the regression using the total COVID-19 pandemic intensity index are similar, as shown in Table A5.

**Table 5:** The effects of COVID-19 on the income of different groups.

	Child’s Income Rank		Parental Income Rank		Child’s Education Years	
	(1)	(2)	(3)	(4)	(5)	(6)
COVID_L × T	−0.135 ***		−0.161 ***		−0.091 ***	
	(0.043)		(0.055)		(0.005)	
COVID_T × T		−0.146 ***		−0.168 **		−0.093 ***
		(0.053)		(0.062)		(0.005)
Child’s IR × COVID_L × T	0.011 ***					
	(0.004)					
Child’s IR × COVID_T × T		0.012 **				
		(0.005)				
Parental IR × COVID_L × T			0.010 ***			
			(0.003)			
Parental IR × COVID_T × T				0.011 ***		
				(0.004)		
Child’s edu year × COVID_L × T					0.006 ***	
					(0.000)	
Child’s edu year × COVID_T × T						0.006 ***
						(0.000)
Constant	5.878 ***	5.882 ***	7.422 ***	7.423 ***	6.128 ***	6.128 ***
	(0.723)	(0.723)	(0.854)	(0.853)	(0.740)	(0.740)
Individual controls	Yes	Yes	Yes	Yes	Yes	Yes
Province FE	Yes	Yes	Yes	Yes	Yes	Yes
Year FE	Yes	Yes	Yes	Yes	Yes	Yes
R-squared	0.453	0.453	0.553	0.553	0.452	0.452
Observations	11,847	11,847	6052	6052	11,847	11,847

Robust standard errors are shown in parentheses; ***, **, and * denote significance at the 1%, 5%, and 10% confidence levels respectively. In all regressions, robust standard errors are clustered at the province level. Individual controls, province fixed effect, and survey year fixed effect are included in all regressions. Both child’s and parental income are divided into 20 equal points within the same generation as income rank variables, increasing from 1 to 20.

## Data Availability

The data used to support the findings of this study are available from the corresponding author upon request.

## Appendix A

See Table A1, Table A2, Table A3, Table A4 and Table A5.

**Table A1:** The relationship between COVID-19 intensity index and major socioeconomic variables.

	Univariate Regressions						Multivariate
	(1)	(2)	(3)	(4)	(5)	(6)	(7)
GDP 2019	0.000						0.000
	(0.000)						(0.000)
Population		0.000					−0.001
		(0.000)					(0.001)
Migration			−0.025				−0.048
			(0.034)				(0.076)
Inter-provincial migration				−0.028			−0.137
				(0.036)			(0.148)
Unemployment rate 2019					−1.032		−1.397
					(1.048)		(1.421)
Urban rate 2019						0.003	0.083
						(0.011)	(0.081)
Constant	0.353	0.420	1.707	1.075	3.841	0.608	3.124
	(0.295)	(0.312)	(1.840)	(0.960)	(3.682)	(1.070)	(3.898)
R-squared	0.011	0.005	0.005	0.008	0.032	0.000	0.111
Observations	31	31	31	31	31	31	31

Robust standard errors are shown in parentheses; ***, **, and * denote significance at the 1%, 5%, and 10% confidence levels respectively. Migration variable includes both intra-provincial and inter-provincial migrants, and inter-provincial migration variable only includes inter-provincial migrants.

**Table A2:** The effects of COVID-19 shock on intergeneration income mobility except for unemployed.

	ln (Child’s Income)		Income Rank	
	(1)	(2)	(3)	(4)
ln (parental income)	0.078 ***	0.078 ***		
	(0.017)	(0.017)		
COVID_L × T	−0.996 ***		−1.062 ***	
	(0.306)		(0.152)	
ln (parental income) × COVID_L × T	0.091 ***			
	(0.027)			
COVID_T × T		−1.034 ***		−1.081 ***
		(0.348)		(0.177)
ln (parental income) × COVID_T × T		0.095 ***		
		(0.031)		
Parental income rank			0.055 ***	0.055 ***
			(0.016)	(0.016)
Parental IR × COVID_L × T			0.059 ***	
			(0.010)	
Parental IR × COVID_T × T				0.061 ***
				(0.012)
Constant	6.325 ***	6.325 ***	2.595	2.600
	(0.825)	(0.825)	(1.803)	(1.804)
Individual controls	Yes	Yes	Yes	Yes
Province FE	Yes	Yes	Yes	Yes
Year FE	Yes	Yes	Yes	Yes
R-squared	0.545	0.545	0.500	0.500
Observations	5712	5712	5712	5712

Robust standard errors are shown in parentheses; ***, **, and * denote significance at the 1%, 5%, and 10% confidence levels respectively. In all regressions, robust standard errors are clustered at the province level. Individual controls, province fixed effect, and survey year fixed effect are included in all regressions.

**Table A3:** Robustness: using COVID-19 intensity index calculated form June and July pandemic data.

	June		July	
	ln (Child’s Income)	Income Rank	ln (Child’s Income)	Income Rank
	(1)	(2)	(3)	(4)
ln (parental income)	0.074 ***		0.074 ***	
	(0.016)		(0.016)	
COVID_T_June_ × T	−1.010 **	−1.001 ***		
	(0.369)	(0.172)		
ln (parental income) × COVID_ T_June_ × T	0.092 ***			
	(0.033)			
Parental income rank		0.051 ***		0.051 ***
		(0.014)		(0.014)
Parental IR × COVID_T_June_ × T		0.057 ***		
		(0.011)		
COVID_T_July_ × T			−1.018 **	−1.001 ***
			(0.378)	(0.170)
ln (parental income) × COVID_T_July_ × T			0.093 **	
			(0.034)	
Parental IR × COVID_T_July_ × T				0.057 ***
				(0.011)
Constant	6.423 ***	2.502	6.423 ***	2.503
	(0.807)	(1.747)	(0.807)	(1.747)
Individual controls	Yes	Yes	Yes	Yes
Province FE	Yes	Yes	Yes	Yes
Year FE	Yes	Yes	Yes	Yes
R-squared	0.557	0.508	0.557	0.508
Observations	5712	5712	5712	5712

Robust standard errors are shown in parentheses; ***, **, and * denote significance at the 1%, 5%, and 10% confidence levels respectively. In all regressions, robust standard errors are clustered at the province level. Individual controls, province fixed effect, and survey year fixed effect are included in all regressions.

**Table A4:** Placebo tests using the total COVID-19 pandemic intensity index.

	S1: Assumed COVID-19 Year 2018		S2: Assumed COVID-19 Year 2016–2018	
	ln (Income)	Income Rank	ln (Income)	Income Rank
	(1)	(2)	(3)	(4)
ln (parental income)	0.074 ***		0.074 ***	
	(0.016)		(0.016)	
COVID_T × T	0.012	0.226 **	2.162	0.256 *
	(0.265)	(0.108)	(1.488)	(0.131)
ln (parental income) × COVID_T × T	0.002		−0.201	
	(0.025)		(0.142)	
Parental income rank		0.049 ***		0.049 ***
		(0.014)		(0.014)
Parental IR × COVID_T × T		−0.011 *		−0.012
		(0.006)		(0.008)
Constant	6.705 ***	4.073 **	6.713 ***	4.079 **
	(0.848)	(1.713)	(0.847)	(1.713)
Individual controls	Yes	Yes	Yes	Yes
Province FE	Yes	Yes	Yes	Yes
Year FE	Yes	Yes	Yes	Yes
R-squared	0.554	0.526	0.554	0.526
Observations	5724	5724	5724	5724

Robust standard errors are shown in parentheses; ***, **, and * denote significance at the 1%, 5%, and 10% confidence levels respectively. In all regressions, robust standard errors are clustered at the province level. Individual controls, province fixed effect, and survey year fixed effect are included in all regressions.

**Table A5:** Including family- and province-level confounders with total COVID-19 intensity index.

	Univariate Confounder Regression					Multivariate
	(1)	(2)	(3)	(4)	(5)	(6)
ln (parental income)	0.066 ***	0.052 ***	0.095 ***	0.104 ***	0.096 ***	0.100 ***
	(0.020)	(0.017)	(0.022)	(0.019)	(0.016)	(0.026)
COVID_T × T	−1.012 **	−0.932 **	−1.116 ***	−0.995 **	−0.962 **	−1.011 **
	(0.369)	(0.389)	(0.376)	(0.365)	(0.361)	(0.384)
ln (parental income) × COVID_T × T	0.092 ***	0.085 **	0.102 ***	0.091 ***	0.088 **	0.092 **
	(0.033)	(0.035)	(0.034)	(0.033)	(0.032)	(0.035)
ln (parental income) × male	0.011					0.013
	(0.016)					(0.017)
ln (parental income) × father’s hukou		0.029 **				0.016
		(0.012)				(0.011)
ln (parental income) × father’s edu year			−0.003 **			−0.003 *
			(0.002)			(0.001)
ln (parental income) × GDP_2019				−0.000 *		−0.000
				(0.000)		(0.000)
ln (parental income) × migration rate					−0.003 **	−0.002 *
					(0.001)	(0.001)
Constant	6.472 ***	6.520 ***	6.502 ***	6.415 ***	6.454 ***	6.641 ***
	(0.813)	(0.821)	(0.758)	(0.817)	(0.831)	(0.814)
Individual controls	Yes	Yes	Yes	Yes	Yes	Yes
Province FE	Yes	Yes	Yes	Yes	Yes	Yes
Year FE	Yes	Yes	Yes	Yes	Yes	Yes
R-squared	0.557	0.557	0.566	0.558	0.558	0.566
Observations	6052	6044	5871	6052	6052	5863

Robust standard errors are shown in parentheses; ***, **, and * denote significance at the 1%, 5%, and 10% confidence levels respectively. In all regressions, robust standard errors are clustered at the province level. Individual controls, province fixed effect, and survey year fixed effect are included in all regressions.
